# A randomized trial of the effects of the noble gases helium and argon on neuroprotection in a rodent cardiac arrest model

**DOI:** 10.1186/s12883-016-0565-8

**Published:** 2016-04-04

**Authors:** Patrick Zuercher, Dirk Springe, Denis Grandgirard, Stephen L. Leib, Marius Grossholz, Stephan Jakob, Jukka Takala, Matthias Haenggi

**Affiliations:** Department of Intensive Care Medicine, University Hospital – Inselspital and University of Bern, Freiburgstrasse, CH 3010 Bern, Switzerland; Neuroinfection Laboratory, Institute for Infectious Diseases, University of Bern, Friedbühlstrasse 51, CH 3010 Bern, Switzerland

**Keywords:** Cardiac arrest, Resuscitation, Helium, Argon, Rat model

## Abstract

**Background:**

The noble gas xenon is considered as a neuroprotective agent, but availability of the gas is limited. Studies on neuroprotection with the abundant noble gases helium and argon demonstrated mixed results, and data regarding neuroprotection after cardiac arrest are scant. We tested the hypothesis that administration of 50 % helium or 50 % argon for 24 h after resuscitation from cardiac arrest improves clinical and histological outcome in our 8 min rat cardiac arrest model.

**Methods:**

Forty animals had cardiac arrest induced with intravenous potassium/esmolol and were randomized to post-resuscitation ventilation with either helium/oxygen, argon/oxygen or air/oxygen for 24 h. Eight additional animals without cardiac arrest served as reference, these animals were not randomized and not included into the statistical analysis. Primary outcome was assessment of neuronal damage in histology of the region I of hippocampus proper (CA1) from those animals surviving until day 5. Secondary outcome was evaluation of neurobehavior by daily testing of a Neurodeficit Score (NDS), the Tape Removal Test (TRT), a simple vertical pole test (VPT) and the Open Field Test (OFT). Because of the non-parametric distribution of the data, the histological assessments were compared with the Kruskal–Wallis test. Treatment effect in repeated measured assessments was estimated with a linear regression with clustered robust standard errors (SE), where normality is less important.

**Results:**

Twenty-nine out of 40 rats survived until day 5 with significant initial deficits in neurobehavioral, but rapid improvement within all groups randomized to cardiac arrest. There were no statistical significant differences between groups neither in the histological nor in neurobehavioral assessment.

**Conclusions:**

The replacement of air with either helium or argon in a 50:50 air/oxygen mixture for 24 h did not improve histological or clinical outcome in rats subjected to 8 min of cardiac arrest.

## Background

Cardiac arrest is an important cause of global cerebral ischemia and only a minority of resuscitated patients survive in good neurological condition [1–3]. Neuronal injury is not uniformly distributed, and follows a distinct time course with delayed disintegration of neurons [4]. This late neuronal degeneration theoretically opens a window of opportunity to mitigate the devastating effects of ischemia on the brain. Although many substances affecting neuronal inflammation have neuroprotective properties in in-vitro and animal experiments [5, 6], the only neuroprotective measure known to have a profound effect on survival and functional outcome after CA in humans is temperature management [7–9].

The inert gas xenon is known for its anesthetic properties since 70 years [10], and it displays neuroprotective effects in vitro and in different animal models of neuronal injury [10–15]. The exact molecular mechanisms for neuroprotective properties of xenon are not fully understood and probably multifactorial; xenon modulates neuroinflammation and apoptosis, it induces hypoxia-inducible factor-1alpha (HIF-1alpha) it activates TREK-1 channels, and modulates adenosine triphosphate (ATP)-sensitive potassium channels (K_ATP_ channels) [10]. Unfortunately, xenon is not readily available. The more obtainable noble gases helium and argon have also revealed similar properties [16]. In vitro models demonstrated beneficial effects for helium regarding neuron survival in traumatic brain injury [17]. Similar effects are shown for argon in traumatic brain injury [14, 18] and in addition in hypoxia [18, 19] but not in stroke [20].

In vivo, helium reduces the ischemic burden in a combined hypoxia/ligation model [21], but no animal or human data exist to date regarding the effectiveness of helium neuroprotection after cardiac arrest. A recent trial with helium treatment in patients after cardiac arrest suggests that this treatment is safe and feasible in the clinical setting [22]. Regarding argon, animals studies have demonstrated beneficial properties on neuroprotection in stroke [20, 23], in neonatal asphyxia [21] and in cardiac arrest in rats [24–26] and pigs [27]. In the aforementioned rat experiments, animals exposed to 40–70 % argon after cardiac arrest demonstrated less neurologic dysfunction and less injury in histopathological analysis, even after delayed argon exposure. In the porcine experiments, the six animals subjected to a 4 h treatment with 70 % argon displayed significantly less neurological deficits, less increase in neuron-specific enolase and less histological brain injury.

Based on these promising results, and given the theoretical benefits and potential implications in humans, our objective was to investigate the effects of these gases in our rat cardiac arrest model [28]. We hypothesized that the inert gases helium and argon lessen neurological damage after cardiac arrest in an 8 min cardiac arrest model in rats. Neurological damage was defined clinically and histologically. We focused on the hippocampus CA1 region which is considered the most vulnerable area of the brain to hypoxia [29].

## Methods

This animal study was approved by the Animal Care and Experimentation Committee of the Canton of Bern (Nr BE 6/13), Switzerland and followed the Swiss national guidelines for the performance of animal experiments. Forty-eight adult male Wistar rats (9–10 weeks old) were obtained from a commercial breeder (Janvier Labs, Le Genest-Saint-Isle, France) and housed at the central animal facilities of the University.

### Randomisation/group assignment

Before anaesthesia, the rats were randomized to one of the experimental groups (helium, argon, air) by drawing concealed lots. Animals which did not survive surgery or did not have a return of spontaneous circulation (ROSC) were replaced immediately with an animal of the same group. Eight non-ischemic sham animals were added to the study to overview histology and performance in the neurobehavioral tests after anesthesia and surgery only. These animals required a different postoperative care and were thus not randomized and were not included into the statistics. Results of these animals are presented but highlighted as not randomized.

All animals underwent the same anaesthesia and instrumentation procedures and, except the non-ischemic sham animals, experienced cardiac arrest/resuscitation with a standardized protocol [28, 30]. The postoperative proceedings (including postoperative pain medication) were the same in all groups except in the non-ischemic sham animals, which were weaned from mechanical ventilation earlier. The groups were planned as following:

- Group helium: 8-min cardiac arrest, treatment 24 h with a mixture of 50 % helium in oxygen.
- Group argon: 8-min cardiac arrest, treatment 24 h with a mixture of 50 % argon in oxygen.
- Group air: 8-min cardiac arrest, standard treatment 24 h with oxygen titrated to reach a 50 % oxygen in nitrogen gas mix.

### Cardiac arrest and resuscitation protocol

The model and detailed experimental procedures were described elsewhere [28], here we provide the overview.

Anaesthesia was induced with sevoflurane and fentanyl, then orotracheal intubation and mechanical ventilation, followed by surgical insertion of a PE50 catheter into the jugular vein and femoral artery. The animals were paralyzed with vecuronium and cardiac arrest initiated with a mixture of potassium and esmolol. After 8 min resuscitation we started with manual chest compression (two finger approach using the index and middle finger) at a rate of 220 per minute guided by a metronome; and intravenous epinephrine and calcium. Ventilation was adapted according to arterial blood gas analysis (aBGA) after 5 and 15 min post-return of spontaneous circulation (ROSC). Fifteen minutes post ROSC, ventilation was switched to the gas mixture according the group assignment and a final blood gas analysis was performed at ROSC + 60 min before catheter removal and wound closure. Sevoflurane was added at a minimal dose to avoid self-extubation and the animals received fentanyl every hour for 3 h. 3.5 h post ROSC buprenorphine was administered subcutaneously, 4 h post-ROSC the mechanical ventilation was weaned. The non-ischemic sham animals had their wounds closed after the ROSC +15 min blood gas analysis and were weaned from mechanical ventilation thereafter, thus they received buprenorphine only. The rational for this deviation in the non-ischemic sham group is based on the assumption that the higher amount of sevoflurane might have a impact on apoptosis itself [31, 32], even though we would not expect any neuronal damage in sham operated animals.

After extubation, the animals were placed in a custom constructed air-tight single cage on a heating pad for the next 20 h. Argon and helium was purchased in cylinders via hospital pharmacy (Carbagas Switzerland) and mixed with oxygen from the wall outlet. The oxygen concentration in the delivered gas mix was monitored with the gas module of the Datex S-5 anesthesia monitor (Datex Ohmeda, now GE, Helsinki, Finland). The respective gas-mix passed through a heat-exchanger and was maximally moistured and entered the cage with 32 °C at a flow of 1.5 l/min. The animals had full access to water and food in this special cage. Twenty-four hours after ROSC, the animals returned to their standard cage with access to water and food ad libitum.

Five days after cardiac arrest, the animals were euthanized with an overdose of pentobarbital intraperitoneally (150 mg/kg). Then the animal was transcardially perfused via the left ventricle with 150 ml of buffered formalin 4 % in phosphate-buffered saline (PBS) for subsequent decapitation and removal of the brain for dissection of the hippocampus for final preservation.

### Assessments

Determination of neuronal damage was performed on a representative 10 μm section of the hippocampal cornus ammoni 1 (CA1) segment using Fluoro-Jade B (FJB, Millipore) staining. FJB staining allows the identification of degenerating neurons [29]. Fluoro-Jade results were expressed by normalizing the total amount of positive cells by the length of the investigated CA1 segment.

Injury to the hippocampus was also determined by histomorphometry of four representative sections of dorsal (septal pole) CA1 slides stained with cresyl violet. The percentage of cells with apoptotic morphology (shrunken appearance and condensed nucleus) was estimated in the CA1 region by visual observation under a bright-field microscope. Atrophy of the CA1 region, resulting from neuronal loss, was determined using the software ImageJ 1.45 l (National Institutes of Health, USA, http://imagej.nih.gov/ij). For this aim, the area of the CA1 subfield stained by cresyl violet was determined with the software. To correct for differences in the length the CA1 between sections, normalization was performed by dividing the calculated surface by the length of the structure, determined at its border proximal to the dentate gyrus, hence providing an average breadth.

All histopathological evaluations were performed by the same investigator, blinded to the clinical and treatment data of the respective animal.

As secondary outcome we assessed general health and neurological function daily from baseline (pre-arrest) until day 5 using several tests: NDS [33] assessing general behaviour deficits (consciousness, respiration) as well as cranial nerve, motor, sensory and coordination function deficits. In addition, a simple VPT [34, 35] was performed, assessing forelimb strength, ability to grasp and balance as well as the TRT [36], which is considered a sensitive test evaluating sensorimotor integration, originally used to lateralize sensorimotor deficits and adopted and modified to record the time period until removal of both tapes for quantifying neuronal damage.

To determine general activity levels and exploration habits the OFT was used [37]: the animals were placed in the middle of the arena and allowed to freely move for 4 min while being recorded. We analysed the footage, mobility/immobility ratio, extern/intern ratio and the rears (vertical activity).

### Statistics

More than 50 % of the data were distributed non-normal, as assessed by the Shapiro-Wilk test. Data are presented as medians and interquartile ranges [IQR]. Histological assessments were compared with the non-parametric Kruskal–Wallis test. For secondary outcomes, we compared the baseline characteristics using non-parametric statistics (Mann–Whitney *U* test, Kruskal–Wallis test). Because of the non- parametric distribution of the data, the treatment effect in repeated measured assessments could not be examined with a mixed ANOVA. We therefore used a linear regression with clustered robust standard errors (SE), where normality is less important (because of the robust SE). The time effect was assessed with an additional fractional polynomial regression.

As already outlined, the non-ischemic sham animals were not randomized and thus not included in the main analysis. We have included the non-ischemic sham animals in the tables to show reference values, and added post-hoc non-parametric tests (Friedman, Wilcoxon) for the non-randomized and randomized groups. These additional values are printed in italics to indicate them as non-randomized and post-hoc, and these values should be interpreted cautiously.

A *p* < 0.05 was considered as significant. Standard calculations were performed using SigmaPlot/SigmaStat 12.5 (Systat Software GmbH, Erkrath, Germany), linear regression with robust clustered SE and fractional polynominal regression was performed with STATA 13 (StataCorp; College Station, TX, USA).

## Results

**Fig. 1:**
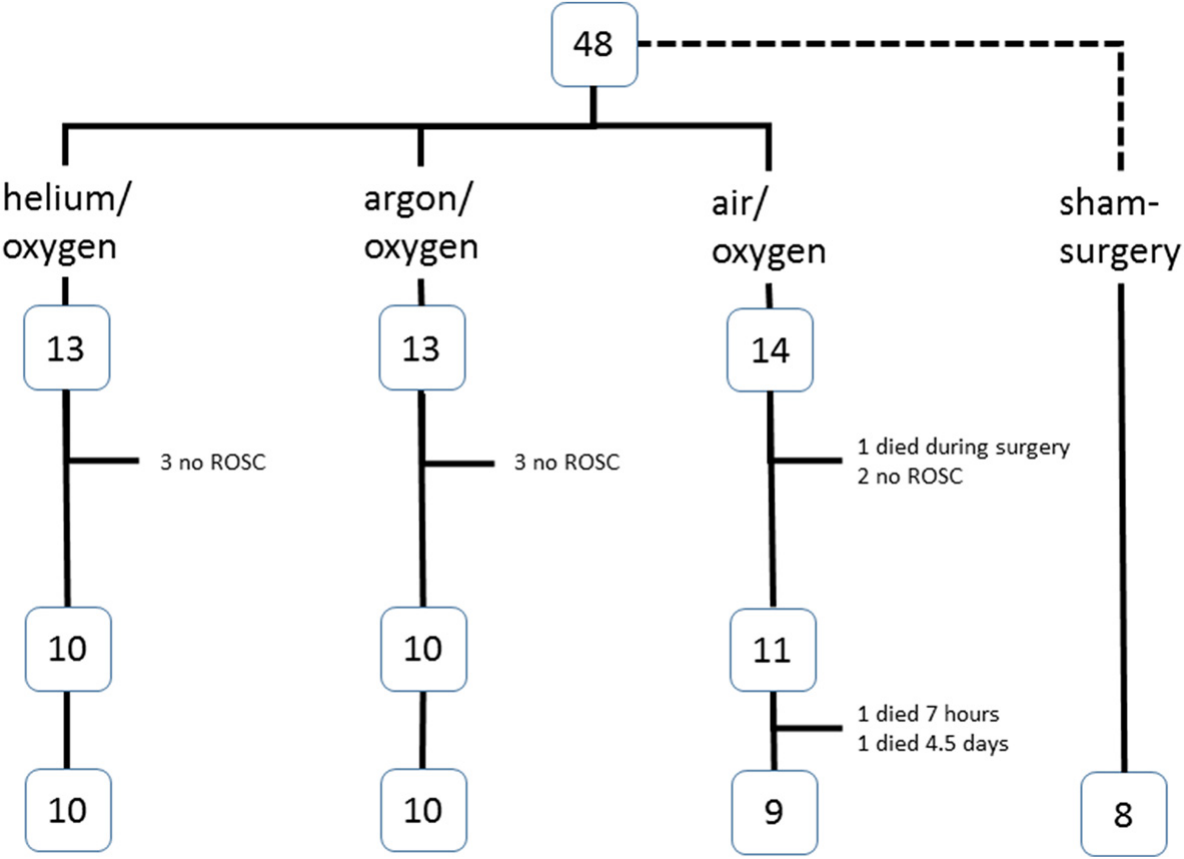
Flow chart of the study. The non-ischemic sham animals were not randomized and were not subjected to cardiac arrest, therefore they were not included into statistics

Of the 40 animals which were randomized one animal died due to surgical complications, and 39 animals entered the cardiac arrest protocol. Of these 39 animals 31 reached a ROSC (82 %), one animal died 7 h post ROSC, another one 4.5 days after ROSC and after initial recovery, both animals were in the air group (Fig. 1).

Baseline characteristics of all animals in terms of haemodynamics (heart rate, mean arterial pressure (MAP), metabolics/respiration (glucose, lactate, pH, pO2, pCO2), temperature and weight did not differ between groups (Tables 1 and 2). Time to ROSC did not significantly differ between the groups (helium 59 s [42–65], air 59 s [45–76] and argon 70 s [55–83], *p* = 0.25).

**Table 1:** Hemodynamic data and blood gas analysis

		Helium/oxygen	Argon/oxygen	Air/oxygen	*Kruskall - Wallis*	*Non-ischemic sham*
Mean Arterial Pressure (MAP)^a^ [mmHg]	Baseline (Before)	100 [86–114]	99 [90–108]	100 [82–118]	*p* = 1.00	*90 [79–92]*
	5 min	125 [115–137]	121 [109–138]	123 [109–129]		*88 [78–102]*
	15 min	76 [62–94]	79 [77–92]	85 [69–100]		*98 [87–105]*
	30 min	85 [65–98]	73 [68–86]	85 [66–93]		*NA*
	60 min	95 [82–97]	85 [67–101]	99 [83–111]		*NA*
*Friedman-Test p*		*<0.001* ^a^	*<0.001* ^a^	*0.004* ^a^		*0.964*
Linear regression with clustered robust SE *p* = 0.621						
(Coef. helium: −1.93 CI −11.49 to 7.63; Coef. argon: −4.71 CI −14.70 to 5.28)						
Time effect: fractional polynomial regression *p* < 0.001						
Heart rate^a^ [1 min^−1^]	Baseline (Before)	300 [268–345]	340 [298–383]	360 [325–395]	*p* = 0.10	*300 [285–380]*
	5 min	300 [288–325]	290 [270–330]	320 [265–355]		*320 [320–380]*
	15 min	300 [285–325]	310 [290–340]	290 [280–340]		*360 [340–380]*
	30 min	360 [320–400]	320 [308–410]	395 [345–420]		*NA*
	60 min	340 [300–400]	340 [323–435]	420 [375–450]		*NA*
*Friedman-Test p*		*0.058*	*0.005* ^a^	*<0.001* ^a^		*0.369*
Linear regression with clustered robust SE *p* = 0.056						
(Coef. helium: −30.3 CI −56.0 to −4.7; Coef. argon: −17.3 CI −50.1 to 15.5)						
Time effect: fractional polynomial regression *p* < 0.001						
Glucose^a^ [mmol/l]	Baseline (Before)	10.5 [8.9–11.6]	9.9 [8.8–10.9]	10.4 [8.4–11.1]	*p* = 0.87	*9.8 [8.7–11.2]*
	5 min	13.7 [13.4–14.5]	13.3 [11.1–16.0]	13.5 [13.1–15.6]		*8.2 [7.5–10.2]*
	15 min	12.5 [11.5–13.5]	13.5 [11.2–14.3]	13.7 [10.6–14.8]		*9.0 [7.0–10.6]*
	60 min	5.7 [4.3–6.6]	5.8 [5.1–6.4]	5.7 [5.3–6.1]		*NA*
*Friedman-Test p*		*<0.001* ^a^	*<0.001* ^a^	*<0.001* ^a^		*0.12*
Linear regression with clustered robust SE *p* = 1.000						
(Coef. helium: 0.06 CI −1.55 to 1.67; Coef. argon: 0.04 CI −1.63 to 1.70)						
Time effect: fractional polynomial regression *p* < 0.001						
Lactate^a^ [mmol/l]	Baseline (Before)	1.2 [0.9–1.3]	1.2 [1.0–1.7]	1.1 [1.0–1.5]	*p* = 0.72	*1.0 [0.9–1.4]*
	5 min	7.0 [6.4–7.5]	7.2 [6.1–8.2]	6.8 [6.3–8.0]		*1.0 [0.6–1.2]*
	15 min	3.5 [3.2–3.8]	3.2 [3.0–3.9]	3.6 [3.0–4.4]		*0.9 [0.6–1.8]*
	60 min	0.8 [0.6–1.2]	0.7 [0.6–0.8]	0.7 [0.6–0.8]		*NA*
*Friedman-Test p*		*<0.001* ^a^	*<0.001* ^a^	*<0.001* ^a^		*0.531*
Linear regression with clustered robust SE *p* = 0.771						
(Coef. helium: 0.38 CI −0.81 to 1.57; Coef. argon: −0.04 CI −0.56 to 0.48)						
Time effect: fractional polynomial regression *p* < 0.001						
pH^a^	Baseline (Before)	7.50 [7.45–7.53]	7.50 [7.45–7.54]	7.42 [7.40–7.49]	*p* = 0.17	*7.41 [7.37–7.51]*
	5 min	7.09 [7.01–7.15]	7.02 [6.96–7.10]	7.09 [6.98–7.16]		*7.44 [7.36–7.47]*
	15 min	7.24 [7.16–7.28]	7.16 [7.08–7.26]	7.30 [7.10–7.31]		*7.45 [7.347–7.49]*
	60 min	7.38 [7.32–7.44]	7.35 [7.30–7.39]	7.39 [7.33–7.41]		*NA*
*Friedman-Test p*		*<0.001* ^a^	*<0.001* ^a^	*<0.001* ^a^		*0.967*
Linear regression with clustered robust SE *p* = 0.631						
(Coef. helium: 0.11 CI −0.37 to 0.15; Coef. argon: −0.02 CI −0.08 to 0.05)						
Time effect: fractional polynomial regression *p* = 0.037						
pO_2_ [mmHg]	Baseline (Before)	114 [106–124]	114 [101–132]	106 [83–139]	*p* = 0.83	*83 [74–97]*
	5 min	203 [146–246]	155 [127–250]	195 [265–355]		*332 [270–342]*
	15 min	125 [101–159]	109 [105–143]	129 [118–165]		*240 [90–324]*
	60 min	159 [136–185]	140 [128–181]	195 [182–212]		*NA*
*Friedman-Test p*		*0.007* ^a^	*<0.001* ^a^	*<0.001* ^a^		*0.236*
Linear regression with clustered robust SE *p* = 0.373						
(Coef. helium: −45.5 CI −114.2 to 23.2; Coef. argon: −48.8 CI −118.9 to 21.3)						
Time effect: fractional polynomial regression *p* = 0.670						
pCO_2_^a^ [mmHg]	Baseline (Before)	32 [29–37]	31 [27–35]	37 [27–39]	*p* = 0.69	*34 [32–42]*
	5 min	46 [38–66]	53 [40–66]	46 [37–52]		*35 [32–43]*
	15 min	39 [36–53]	44 [35–62]	36 [32–46]		*36 [30–43]*
	60 min	39 [34–47]	41 [35–49]	35 [31–39]		*NA*
*Friedman-Test p*		*0.002* ^a^	*0.003* ^a^	*0.013* ^a^		*0.335*
Linear regression with clustered robust SE *p* = 0.741						
(Coef. helium: 2.07 CI −6.03 to 10.17; Coef. argon: 3.40 CI −5.80 to 12.59)						
Time effect: fractional polynomial regression *p* < 0.001						
Temp. [°C]	Baseline (Before)	36.0 [35.7–36.2]	36.4 [36.1–36.5]	36.0 [35.8–36.2]	*p* = 0.07	*36.0 [35.8–37.1]*
	5 min	35.3 [35.0–35.4]	35.0 [34.8–35.6]	35.3 [34.7–35.6]		*36.5 [36.0–36.9]*
	15 min	36.2 [36.0–36.3]	36.2 [35.8–36.2]	36.2 [36.0–36.6]		*36.4 [36.1–36.6]*
	60 min	36.8 [36.3–37.4]	36.6 [36.4–37.2]	37.0 [36.7–37.3]		*NA*
*Friedman-Test p*		*<0.001* ^a^	*<0.001* ^a^	*0.002* ^a^		*0.967*
Linear regression with clustered robust SE *p* = 0.378						
(Coef. helium: 0.42 CI −0.54 to 1.37; Coef. argon: −0.84 CI −2.46 to 0.77)						
Time effect: fractional polynomial regression *p* = 0.487						

Comparisons of baseline parameters were calculated with the Kruskal-Wallis ANOVA on Ranks. Differences between the groups were computed with a linear regression with clustered robust SE, time effect with fractional polynomial regression. The animals in the non-ischemic sham group were not randomized, and thus indicated in italic style. These animals are not included in the main statistical analysis. The post-hoc Kruskall Wallis and Friedman tests were performed to compare groups at baseline and the time effect within the groups, but should be interpreted with caution (see text). Data are given as median and [interquartile ranges]

^a^ denotes significant time effects

**Table 2:** Results of neurobehavioral tests

		Helium/oxygen	Argon/oxygen	Air/oxygen	*Kruskall - Wallis*	*Non-ischemic sham*
Weight^a^ [g]	Baseline (Before)	358 [343–383]	343 [334–367]	345 [326–372]	*p* = *0.375*	*400 [375–413]*
	Day 1	342 [328–367]	335 [314–361]	339 [322–360]		*384 [360–409]*
	*Wilcoxon Test p*	*0.01*	*<0.01*	*0.02*		*0.04*
	Day 2	338 [322–354]	323 [306–351]	323 [304–345]		*389 [365–404]*
	Day 3	342 [327–362]	327 [306–355]	330 [308–343]		*391 [361–408]*
	Day 4	339 [327–372]	329 [309–365]	328 [313–345]		*400 [369–410]*
	Day 5	348 [324–380]	341 [319–370]	332 [318–355]		*401 [374–415]*
*Friedman-Test p*		*<0.001* ^a^	*<0.001* ^a^	*<0.001* ^a^		*<0.001* ^a^
Linear regression with clustered robust SE *p* = 0.302						
(Coef. helium: −11.9 CI −33.0 to 9.2; Coef. argon: −15.2 CI −36.1 to 5.6)						
Time effect: fractional polynomial regression *p* = 0.001						
Tape Removal Test^a^ [seconds]	Baseline (Before)	18 [11–29]	9 [5–12]	13 [8–23]	*p* = *0.06*	*17 [14–39]*
	Day 1	74 [21–120]	120 [35–120]	85 [23–120]		*10 [8–21]*
	*Wilcoxon Test p*	0.01	<0.01	<0.01		*0.38*
	Day 2	21 [12–76]	18 [11–111]	17 [9–120]		*14 [7–18]*
	Day 3	20 [8–113]	16 [11–120]	31 [9–88]		*18 [15–95]*
	Day 4	14 [10–113]	11 [7–59]	17 [9–120]		*19 [7–96]*
	Day 5	12 [7–45]	9 [5–44]	11 [7–93]		*12 [7–17]*
*Friedman-Test p*		*0.018* ^a^	*0.002* ^a^	*0.023* ^a^		*0.086*
Linear regression with clustered robust SE *p* = 0.980						
(Coef. helium: −2.01 CI −35.88 to 31.86; Coef. argon: −3.24 CI −36.49 to 30.01)						
Time effect: fractional polynomial regression *p* < 0.001						
Neuro Deficit Score^a^ [range: 0–100]	Baseline (Before)	0 [0–0]	0 [0–0]	0 [0–0]	*p* = 0.37	0 [0–0]
	Day 1	2.5 [0–3.0]	3.0 [0.5–3.0]	2.5 [0.5–3.0]		0 [0–0]
	*Wilcoxon Test p*	0.02	0.02	0.03		1.0
	Day 2	0 [0–2.3]	0 [0–2.0]	0 [0–1.5]		0 [0–0]
	Day 3	0 [0–0.3]	0 [0–1.3]	0 [0–0.5]		0 [0–0]
	Day 4	0 [0–0.3]	0 [0–0.5]	0 [0–0]		0 [0–0]
	Day 5	0 [0–0.3]	0 [0–0.3]	0 [0–0]		0 [0–0]
Friedman-Test p		<0.001^a^	<0.001^a^	<0.001^a^		1.0
Linear regression with clustered robust SE *p* = 0.612						
(Coef. helium: 0.50 CI −0.68 to 1.69; Coef. argon: 0.35 CI −0.63 to 1.32)						
Time effect: fractional polynomial regression *p* < 0.001						
Vertical Pole Test^a^ [range: 0–3]	Baseline (Before)	0 [0–0]	0 [0–0]	0 [0–0]	*p* = 0.60	0 [0–0]
	Day 1	2.5 [0.25–3.0]	3.0 [1.0–3.0]	2.5 [1.5–3.0]		0 [0–0]
	*Wilcoxon Test p*	0.03	0.02	0.03		1.0
	Day 2	0 [0–2.0]	0 [0–1.5]	0 [0–1.0]		0 [0–0]
	Day 3	0 [0–0]	0 [0–1.0]	0 [0–0]		0 [0–0]
	Day 4	0 [0–0]	0 [0–0]	0 [0–0]		0 [0–0]
	Day 5	0 [0–0]	0 [0–0]	0 [0–0]		0 [0–0]
Friedman-Test p		<0.001^a^	<0.001^a^	<0.001^a^		1.0
Linear regression with clustered robust SE *p* = 0.663						
(Coef. helium: 0.15 CI −0.34 to 0.64; Coef. argon: 0.21 CI −0.32 to 0.75)						
Time effect: fractional polynomial regression *p* < 0.001						
Open Field Test: distance^a^ [cm]	Baseline (Before)	1691 [1327–1900]	1769 [1620–2101]	1580 [1520–1951]	*p* = 0.23	1693 [1378–1938]
	Day 4	1673 [1161–1919]	1320 [1144–1712]	2058 [1698–2403]		1034 [799–1698]
	Day 5	851 [758–1343]	1240 [658–1606]	962 [711–1629]		774 [698–1196]
Friedman-Test p		0.06	0.07	0.24		0.02^a^
Linear regression with clustered robust SE *p* = 0.156						
(Coef. helium: −244 CI −528 to 39; Coef. argon: −5 CI −465 to 455)						
Time effect: fractional polynomial regression *p* = 0.002						
Open Field Test: mobility/immobility ratio^a^ [%]	Baseline (Before)	82 [77–88]	88 [81–92]	84 [82–87]	*p = 0.24*	*83 [80–85]*
	Day 4	81 [71–84]	81 [75–88]	85 [82–87]		*65 [54–84]*
	Day 5	59 [51–72]	77 [50–86]	67 [46–82]		*56 [41–65]*
*Friedman-Test p*		*0.15*	*0.20*	*0.06*		*<0.01* ^a^
Linear regression with clustered robust SE *p* = 0.112						
(Coef. helium: −4.63 CI −11.87 to 2.62; Coef. argon: 2.51 CI −6.22 to 11.24)						
Time effect: fractional polynomial regression *p* < 0.001						
Open Field Test: extern/intern ratio^a^ [%]	Baseline (Before)	94 [93–96]	94 [93–96]	96 [94–97]	*p = 0.84*	*96 [93–98]*
	Day 4	99 [97–100]	98 [96–99]	95 [95–97]		*100 [97–100]*
	Day 5	100 [99–100]	99 [99–100]	99 [95–100]		*100 [98–100]*
*Friedman-Test p*		*0.02* ^a^	*0.03* ^a^	*0.03* ^a^		*0.03* ^a^
Linear regression with clustered robust SE *p* = 0.501						
(Coef. helium: 0.68 CI −1074 to 2.10; Coef. argon: 0.33 CI −1.55 to 2.22)						
Time effect: fractional polynomial regression *p* = 0.003						
Open Field Test: rears/vertical^a^ [count]	Baseline (Before)	15 [12–23]	20 [19–23]	19 [16–22]	*p = 0.21*	*17 [14–22]*
	Day 4	6 [5–8]	5 [3–10]	8 [5–9]		*9 [7–13]*
	Day 5	3 [1–4]	6 [2–9]	4 [2–7]		*7 [4–9]*
*Friedman-Test p*		*<0.001* ^a^	*<0.001* ^a^	*<0.001* ^a^		*<0.001* ^a^
Linear regression with clustered robust SE *p* = 0.107						
(Coef. helium: −2.73 CI −5.58 to 0.11; Coef. argon: −0.40 CI −4.13 to 3.33)						
Time effect: fractional polynomial regression *p* < 0.001						

Comparisons of baseline were calculated with the Kruskal-Wallis ANOVA on ranks. Differences between the groups were computed with a linear regression with clustered robust SE, time effect with fractional polynomial regression. The animals in the non-ischemic sham group were not randomized, and thus indicated in italic style. These animals are not included in the main statistical analysis. The post-hoc Kruskall Wallis; Wilcoxon and Friedman tests were performed to compare groups at baseline and the time effect within the groups, but should be interpreted with caution (see text). Data are given as median and [interquartile ranges]

^a^ denotes significant time effects

There were no differences in the trends of haemodynamics, metabolics/respiration, temperature or weight between goups. The animals developed a significant lactic acidosis, but without differences between the groups. MAP increased after resuscitation, at 15 min the blood pressure reached a minimum and then started to increase towards baseline pressure again. Only heart rate tended to increase more in the air group compared to the noble gas groups (Table 1, *p* = 0.056).

The results of the TRT, the NDS and the VPT did not differ between the groups (Table 2). In these tests the animals demonstrate significant impairment on day one, and start to recover until they reach baseline levels on day 4–5 (TRT), or day 2 (NDS and VPT).

In the OFT, the resuscitated animals showed a decrease in exploratory behavior between day 4 and day 5, but without significant differences between the groups (Table 2).

**Fig. 2:**
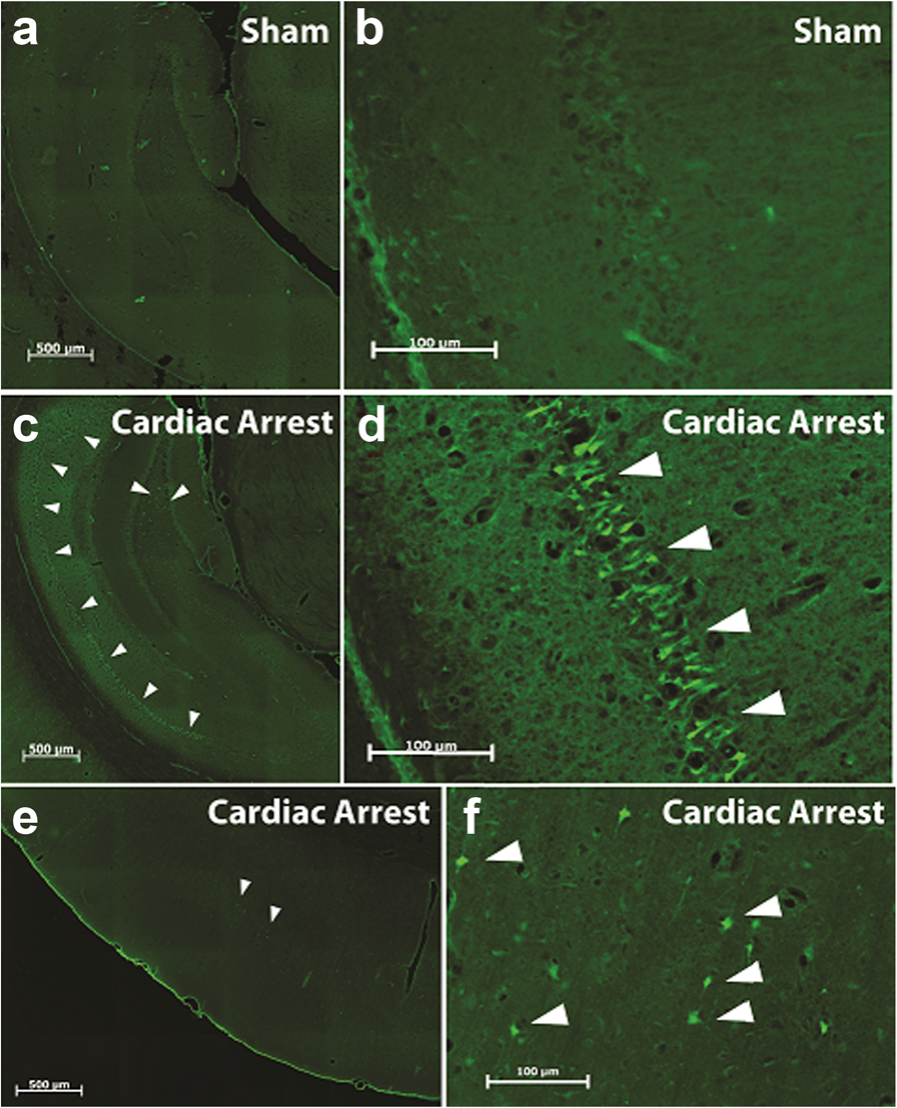
Examples of Fluoro-Jade B staining of the hippocampus. Overview of the hippocampus (**a** and **c**) and close-up view of the CA1 region (**b**) and (**d**) of a sham animal without cardiac arrest (**a** and **b**) and of an animal with cardiac arrest (**c** and **d**). Compared to the animal with surgery only, FJB staining (indicating degenerating neurons, *white arrowheads*) was prominently present in the CA1 region, with occasionally positive cells scattered throughout the hilus region. A few dispersed cells (*white arrowheads*) are found in the cortex of animals with cardiac arrest stain positive for FJB (**e**, overview and **f**, close-up view)

**Fig. 3:**
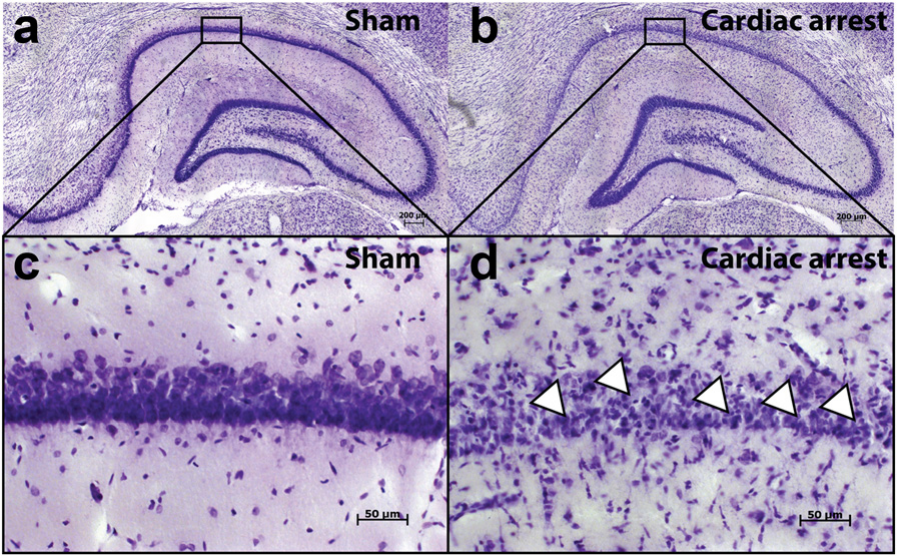
Examples of Cresyl Violet staining of the hippocampus CA1 segment. Overview of the hippocampus (**a** and **b**) and close-up view of the CA1 region (**c**) and (**d**) of a sham animal without cardiac arrest (**a** and **c**) and of an animal with cardiac arrest and argon treatment (**b** and **d**). *Arrowheads* show pyknotic cells, indicating apoptosis in the animal which suffered from cardiac arrest

In the histologic examinations substantial damage could be found in the animals which suffered from cardiac arrest compared to the non-randomized non-ischemic sham animals. FJB staining demonstrated an absence of neuronal degeneration in the non-ischemic sham animals, but no differences between groups in the resuscitated rats (details Table 3 and Fig. 2). In the cresyl violet stained slides, no cells with signs of ischemic damage could be seen in the non-randomized non-ischemic sham animals, as assessed by pyknotic cell counts. Compared to air/oxygen rats with up to 80 % [IQR 61–93] destructed cells, the noble gas treated animals trended to less damage (helium 53 % [24–76], argon 59 % [44–86], *p* = 0.09, Table 3, Fig. 3). The cell layer of the CA1 segment depicted atrophy in the resuscitated animals as determined by a reduced average breadth, but no differences between the noble gases and the air rats (details see Table 3). Only few injured neurons could be seen in the cortex in the FJB (picture 2 E and F). This due to the uneven distribution of neuronal injury after hypoxia, where the neurons of the CA1 layer are considered as the most vulnerable neurons throughout the brain [38, 39].

**Table 3:** Histomorphometry of the hippocampus CA1 segment on day 5

	Helium/oxygen	Argon/oxygen	Air/oxygen	Kruskall - Wallis	*Non-ischemic sham*
Fluoro-Jade [numbers of stained cells per mm]	148 [140–166]	154 [124–199]	145 [138–182]	*p* = 0.83	*0 [0–0]*
pyknotic cells in CA1 [%]	53 [24–76]	59 [44–86]	80 [61–93]	*p* = 0.09	*0 [0–1]*
CA1 cell layer atrophy (normalized: surface/length) [mm^2^/mm]	0.065 [0.058–0.083]	0.074 [0.061–0.080]	0.061 [0.043–0.069]	*p* = 0.11	*0.082 [0.077–0.088]*

Histological assessment of the hippocampus CA1 segment on day 5 shows a substantial damage after cardiac arrest, but there were no differences between the resuscitated animals. The animals in the non-ischemic sham group were not randomized, and thus indicated in italic style. These animals are not included in the main statistical analysis

## Discussion

In our model of 8 min cardiac arrest, the animals suffered from a considerable hypoxic-ischemic event, demonstrated by the important delays for executing the neurobehavioral tasks (Table 2) and the extensive damage in the histological examinations (Table 3). The exposure to helium or argon in 50 % oxygen for 24 h did not ameliorate the extent of the damage: we did not find any significant statistical differences in the neurobehavioral tests and in Fluoro-Jade B staining. In the cresyl violet staining, the histological analysis tends towards less damaged cells in the CA1 segment of the hippocampus in the noble gas groups (helium better than argon), resulting in a more preserved cell layer of the CA1 segment. Looking at the entire cell layer breadth though, argon treated animals have more preserved cells than the rats in the helium group, which is unexpected, regarding the cell count in the resyl violet staining. We believe this difference is based on the difficulties to delineate the borders of the curved CA1 segment exactly in plane microscopy pictures.

Our results are not in line with those recently published by Brücken et al. and by Ristagno et al. in regard of the neuroprotective properties of argon [24, 26, 27], which could be explained by some differences in the experiment settings. In Brücken’s group, the rats sustained a shorter cardiac arrest (7 min vs 8 min in our experiments), but the time from induction of CA to ROSC was about 645 s in their study, compared with 540 [IQR 529 to 560] seconds, a considerable difference in total ischemia time, which might results in a more severe damage. This is further supported by the fact, that Brücken’s animals were more severely impaired in the neuropsychological testing. As for the pigs used in the Ristagno’s experiment, the time period until return of a stable circulation was even longer, with a combined no-flow and low-flow time of 817 s. Further differences affect the argon application: Brücken used a 70 % argon in oxygen mixture and started 60 min post ROSC, the argon was applied for 60 min. Ristagno also used 70 % argon in oxygen, but started 5 min after ROSC and continued for 4 h. The rational for administering 50 % argon early after the insult in our protocol is based on the work by David et al. [20] and Loetscher et al. [18], both found a maximum neuroprotective effect with a 50 % argon 25 % oxygen 25 % nitrogen gas mix. Increasing the argon concentration beyond 50 % reduced the success of neuroprotection in their experiments. Ryang et al. [23] also used 50 % argon 50 % oxygen mix in their model of transient middle cerebral artery occlusion (MCAO), where an important reduction of neuronal damage could be detected.

Different timing of argon application might be important too: Ryang administered the argon during ischemia and before reperfusion [23] and David used a similar approach in the in vivo experiments [20]. Brücken found a significant effect even after a delayed administration of 3 h [26]. Loetscher subjected hippocampal neurons to delayed argon treatment and found a time-dependent reduction of efficacy [18]. Based on these results, we administered the noble gases early after cardiac arrest and thus expected a significant improvement.

Another significant difference between these above mentioned studies affects the duration of gas administration. In our experiment we had a long exposure for 24 h compared to 1 h (Brücken), respective 4 h (Ristagno).

We are unaware of negative effects of prolonged helium or argon administration, and the positive effects found by Loetscher et al. were produced with a 3-day incubation period of the hippocampal slices [18]. Generally, mechanical ventilation with a helium/oxygen mixture (Heliox) is regarded as safe and is used since 1934 without negative effects [40].

With regard to histology, the differences between our results and the results of Brücken need to be mentioned. The hippocampus CA1 region is considered as the most vulnerable area of the brain after hypoxic ischemic injury [38]. As expected, in the Brücken experiments most damage could be found in this specific region, with a neuronal damage index of about 3 (from 0 to 11; 0 indicating no damage, 11 indicating complete destruction) in both (air and argon) groups. The statistically significant difference in neuronal damage between the animals in the air and argon groups were discovered in the less vulnerable neocortex areas and in the CA3/4 segment of the hippocampus with a neuronal damage index of about 1.0 in the air group and 0.7 in the argon group, but there were no significant differences within the CA1 segment between the groups. In a further publication of the model, the CA1 region was even not reported [25, 26]. Different impact of argon neuroprotection between cortex and subcortical areas has also been described elsewhere [20].

Helium as a neuroprotective agent is less investigated and the results are ambiguous: some authors did not find any [14] or only minor positive effects [21], others reported significant positive effects in in vitro experiments [17], whilst other studies found even detrimental effects [19]. In vivo, exposure to helium reduced infarct volume in a rat MCAO model [41] as well as in a model inflicting moderate hypoxic-ischemic brain injury in rat pups [21]. It has been discussed whether these discrepant findings could be explained by compressed helium-induced hypothermia, because helium possesses a high heat-transfer capacity. In a study where helium was warmed up, no neuroprotective capacity could be detected [42].

Limitations of our study include the small numbers of animals per group, which decreases the potential to detect a small difference. This argument is especially important for negative trials at risk for type II errors, when an underpowered study concludes a possible helpful intervention as unusable. A post-hoc sample size calculation with the (false) assumption of normal distribution would indicate a group size of 44 animals per group to detect a 20 % difference in pyknotic cells in the cresyl violet staining and a group size of 114 animals per group for a 20 % difference in the FJB staining (with a power of 0.8 and an alpha of 0.05). The wide confidence intervals in the regression analysis of the neurobehavioral data and the aforementioned post-hoc sample size calculation of the histology assessments data make the possibility of rendering the study “positive” simply by increasing the numbers of animals improbable.

Temperature management could also be a relevant issue. It can be argued that the baseline temperature before cardiac arrest was not exactly the same (without statistical significance). In the argon group we observed a more prominent temperature drop at 5 min post ROSC without being statistically significant comparing to the other groups. And given that hypothermia is neuroprotective, we would have expected a better outcome in the argon group, which was not the case in the present study.

The neurobehavioural tests we used can be criticized as not sensitive enough to detect small but significant differences in a cardiac arrest model. So the neurodeficit score ranges from 0 to 100 (with 100 indicating brain death), and the median value at day 1 was 5 in the argon group, indicating rather mildly affected animals after resuscitation. The dorso-septal pole of the hippocampus in rats is responsible for spatial learning. Performing different tests, e.g. a water-maze or novel object location test [43] might be better tests for testing the damage in this area; with the limitation of a more difficult interpretation of these tests due to different behavioral patterns depending on age after cardiac arrest [44].

Brain regions other than the hippocampus CA1 segment have not been examined in detail. As shown as an example in Fig. 2 in the dentate gyrus (2 C) or cortex (2 E and F), other regions of the brain show only minimal neuronal degeneration, a finding consistent with the literature [38]. Quantification and group comparison of these minimal changes are unlikely to provide reliable statistical data because of the wide dispersal of their numbers.

In this series we have not added immunostaining to elucidate the pathway of neuronal degeneration. We cannot prove that the predominant form of neuronal degeneration is apoptosis because we have not performed staining for Caspase-3 or TUNEL. We know from former experiments that no signs of cell death can be found on day 2 [28], which is an indicator of delayed cell death/apoptosis.

## Conclusion

In conclusion, treatment with neither argon nor helium improved functional outcome or reduced neuronal damage in the most vulnerable part of the brain (hippocampal CA1 region) after 8 min of cardiac arrest. In contrast to in-vitro experiments, helium does not seem to exhibit neuroprotective properties in rats. The population which might profit from noble gas exposure after cardiac arrest, as well as the protocol of administration (timing, duration, concentration), needs to be further characterized.

## Abbreviations

aBGA: arterial blood gas analysis
CA1: region I of hippocampus proper (cornus ammoni)
FJB: fluoro-jade B
MAP: mean arterial pressure
NDS: neuro deficit score
OFT: open field test
ROSC: return of spontaneous circulation
SE: standard error
TRT: tape removal test
VPT: vertical pole test
